# Fast Parameters Estimation in Medication Efficacy Assessment Model for Heart Failure Treatment

**DOI:** 10.1155/2012/608637

**Published:** 2012-10-03

**Authors:** Yinzi Ren, Xiao Fu, Qing Pan, Chengyu Lin, Guiqiu Yang, Li Li, Shijin Gong, Guolong Cai, Jing Yan, Gangmin Ning

**Affiliations:** ^1^Department of Biomedical Engineering, Zhejiang University, Hangzhou 310027, China; ^2^Department of Intensive Care Unit, Zhejiang Hospital, Hangzhou 310013, China

## Abstract

*Introduction*. Heart failure (HF) is a common and potentially fatal condition. Cardiovascular research has focused on medical therapy for HF. Theoretical modelling could enable simulation and evaluation of the effectiveness of medications. Furthermore, the models could also help predict patients' cardiac response to the treatment which will be valuable for clinical decision-making. *Methods*. This study presents a fast parameters estimation algorithm for constructing a cardiovascular model for medicine evaluation. The outcome of HF treatment is assessed by hemodynamic parameters and a comprehensive index furnished by the model. Angiotensin-converting enzyme inhibitors (ACEIs) were used as a model drug in this study. *Results*. Our simulation results showed different treatment responses to enalapril and lisinopril, which are both ACEI drugs. A dose-effect was also observed in the model simulation. *Conclusions*. Our results agreed well with the findings from clinical trials and previous literature, suggesting the validity of the model.

## 1. Introduction

Heart failure (HF) is a serious stage of various heart diseases. High incidence and mortality have made it a significant public health problem around the world [1]. Half of the HF patients die within 4 years, and over half of the patients with severe HF die within 1 year [2]. In the United States, HF is the most common age-related disease, and more medical costs are spent on the diagnosis and treatment of HF than any other diseases [3].

Clinical guidelines, technological developments, and pharmacological interventions have intended to diminish the severity of the disease [4]. With the development in medical science, various hemodynamic parameters have been reported to be vital in HF, such as systolic blood pressure (SBP), diastolic blood pressure (DBP), vascular resistance (*R*), and vascular compliance (*C*). Among them, blood pressure is the most accessible parameter that effectively reflects the overall hemodynamic status. The Framingham Heart Study [5], which was done on 894 men and 1146 women, revealed that the increase of blood pressure is the major risk factor of HF. The study by Gheorghiade et al. identified SBP as an independent predictor of morbidity and mortality in HF [6].

In clinical practice, pharmacological therapy is the main medical treatment for HF. Angiotensin-converting enzyme inhibitors (ACEIs), beta-blockade, and diuretics improve survival in HF patients. However, an optimal therapy dose for a specific individual is hard to determine. Conventionally, large-scale clinical trials are conducted to assess drug efficacy. However, they usually result in a general guide for the whole population, rather than for individuals. In addition, clinical trials involve high costs and long-term efforts. Therefore, modeling and evaluating drug efficacy by mathematical methods have attracted increasing attention.

Such methods are principally based on mathematical models that mimic the behavior of the hemodynamic parameters under medication in the cardiovascular system. Diaz-Insua et al. simulated blood pressure waves by bond graph methods [7]. Ursino and Magosso established a cardiovascular model with arterial baroreceptor, and using that model, the regulation mechanism of acute hemorrhage was simulated [8]. Most cardiovascular models are based on the Windkessel model, constructed by vascular resistance and compliance elements. The resistance and compliance are the primary indicators of the properties of blood vessel, with significant influence on cardiovascular function. Zelis et al. showed that HF may directly increase systemic vascular resistance by altering the mechanical properties and reducing the vasodilation ability of the resistance vessels [9]. Mitchell et al. stated that vessel compliance played a role in the pathophysiology of HF [10]. They explained that the neurohumoral activation increased vascular smooth muscle mass and fibrosis, resulting in decrease of compliance. Under a certain setting of *R* and *C*, the models enabled a simulation of vital physiological parameters, such as SBP and DBP. Tsuruta et al. simulated the HF state and predicted the drug efficacy by setting and adjusting the model parameters [11]. In our study, HF is simulated by raising vascular resistance and reducing vascular capacitance to decrease ventricular contractility and increase diastolic stiffness. The response to medicine therapy is emulated by changing resistance and capacitance parameters simultaneously, which are acquired by solving the model inversely from the measured hemodynamic states. Measurable physiological parameters are used to estimate unknown model parameters and derive the changes of model parameters with respect to doses of a particular drug. Consequently, a specific simulation model for individual patient is achieved to predict the medication effect under different doses. These models make the simulation of optimum dose for individual treatment possible. They also provide virtual cases for clinical experiments, facilitate the investigation of cardiovascular functional mechanism, and give us useful information on medical treatment as well as interpretation.

In order to make the simulated physiological parameters match the real ones, the values of *RC* model parameters must be adjusted by successive approximation. As a consequence, these cardiovascular simulations have a common problem of large computational time in model parameters estimation. In our previous work [12], a mathematical model consisting of 54 mathematical equations is employed to describe the interaction among the whole body cardiovascular circulation. The work indicates that the estimation of model parameters is the time “bottleneck” of the whole simulation process. In the model, *RC* values are reiteratively adjusted to minimize the difference between the simulated hemodynamic parameters (e.g., blood pressure) and the true values, until it lies in an acceptable range. Generally, a single simulation costs 20–30 minutes and it may take 2–5 hours to achieve the appropriate *RC* values. Drug efficacy evaluation and parameters estimation consume 80% of the overall time. For both research and clinical applications, the low efficiency in model parameters estimation needs to be resolved.

The present study aims to address the bottleneck issue of parameter estimation in cardiovascular modeling by developing fast parameters estimation algorithm for pharmacodynamic simulations in predicting HF treatment and individual patient response.

## 2. Methods

In this section, the fast parameter estimation algorithm and pharmacodynamic simulation model are introduced in detail. ACEI is selected in the present study because it is recommended as the first-line therapy in HF patients [1], producing more hemodynamic and symptomatic benefits for the patients than other conventional medicine. The target of ACEI is the resistance and compliance of the vessels, so the effect of such drug can be simulated by adjusting *R* and *C* in a Windkessel model.

### 2.1. Fast Parameter Estimation Algorithm for Cardiovascular Model

In the cardiovascular system model, the model parameters are estimated from physiological parameters directly measured from medical examination. Conventionally, one has to repetitively adjust the model parameters to make the simulated physiological parameters approximate the real ones. Essentially, iteration is a method of enumeration, which consumes a great amount of time and reduces the computation efficiency.

**Figure 1:**
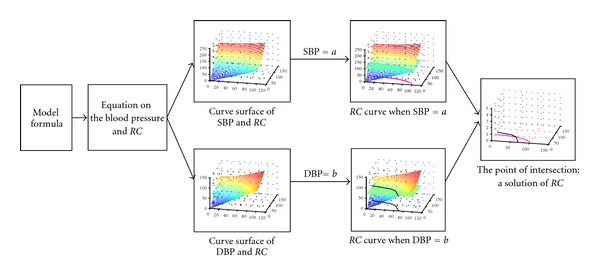
The flow-chart of fast parameters estimation algorithm. The flow-chart shows the process from model formula to the solution of *RC*. The inputs of the process are complex equations and setting conditions. The outputs are the fast estimates of *RC* parameters.

As illustrated in Figure 1, the study proposes a novel method to fastly estimate model parameters by constructing a mapping surface of model parameters and physiological parameters. By inputting a set of measured physiological data, the corresponding model parameters can be estimated quickly on the mapping surface. This fast algorithm cannot only overcome the shortcomings of computational complexity but also make the estimation of model parameters more accurate and reliable. In this study, the inputting data are SBP and DBP, and the outputs are the estimates of vascular resistance and vascular compliance.

The details of the method are described as follows.

**Figure 2:**
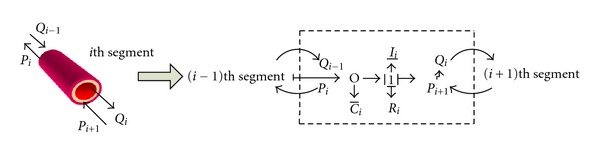
The bond graph of *i*th segment vessel. Bond graph uses several components to represent the real blood vessel. *R*_*i*_, *C*_*i*_, and *I*_*i*_ mean vascular resistance, vascular compliance, and blood inertia, respectively. The “0” crunode indicates the elastic chamber of artery blood vessel. The “1” crunode indicates the artery blood vessel with resistance. For the *i*th segment of blood vessel, the input is the flow *Q*_*i*−1_ of the *i* − 1th vessel segment, which receives the pressure *P*_*i*_ as feedback. The output side transfers the flow *Q*_*i*_ to the *i* + 1th vessel segment and gets the returned pressure *P*_*i*+1_.

The cardiovascular model in this study is constructed by bond graph technique, which uses several components to represent real blood vessel. The “0” crunode indicates the elastic chamber of artery blood vessel and the “1” crunode indicates the artery blood vessel with resistance. The bond graph structure of a vessel segment is shown in Figure 2 and a full description of the model can be found in the literature [12].

The equations corresponding to vascular bond graph are given in [13] as follows:

(1) $$\begin{array}{c} \frac{dV_{i}}{dt}=Q_{i-1}-Q_{i},\\ \frac{d\lambda_{i}}{dt}=P_{i}-P_{i+1}-R_{i}\cdot Q_{i},\\ Q_{i}=\frac{\lambda_{i}}{I_{i}},\\ P_{i}=\frac{V_{i}}{C_{i}}, \end{array}$$

where *P*, *Q*, *λ*, *V*, *R*, *C*, and *I* represent blood pressure, blood flow, pressure momentum, vascular volume, vascular resistance, vascular compliance, and blood inertia, respectively. These four equations can be combined into a second-order differential equation, as in (2):

(2) $$\begin{array}{c} I_{i}C_{i}\frac{d^{2}P_{i}}{dt^{2}}+R_{i}C_{i}\frac{dP_{i}}{dt}+P_{i}=I_{i}\frac{dQ_{i-1}}{dt}+R_{i}Q_{i-1}+P_{i+1}. \end{array}$$

Blood flow *Q*_*i*−1_ is approximated by a sinusoidal function. Assume the cardiac cycle is 0.8 s, and the systolic period is 0.3 s, the input blood flow wave of the model is defined in (3):

(3) $$\begin{array}{c} Q_{i-1}=\{\begin{array}{cc} 70\sin\left(\frac{\pi t}{0.3}\right), & 0\;\text{s}\leq t\leq 0.075\;\text{s},\\ 70\sin\left(\frac{\pi }{4}\right)+\frac{70}{12}\sin\left[\frac{2\pi \left(t-\left(0.3/4\right)\right)}{0.3}\right], & 0.075\;\text{s}\leq t\leq 0.225\;\text{s},\\ 70\sin\left(\frac{\pi t}{0.3}\right), & 0.225\;\text{s}\leq t\leq 0.3\;\text{s},\\ 0, & 0.3\;\text{s}\leq t\leq 0.8\;\text{s}. \end{array} \end{array}$$

By substituting *Q*_*i*−1_ in (2) with the expression of (3), a general solution of the blood pressure in systole period (*t* ∈ [0.075,0.225]) is derived as

**Figure 3:**
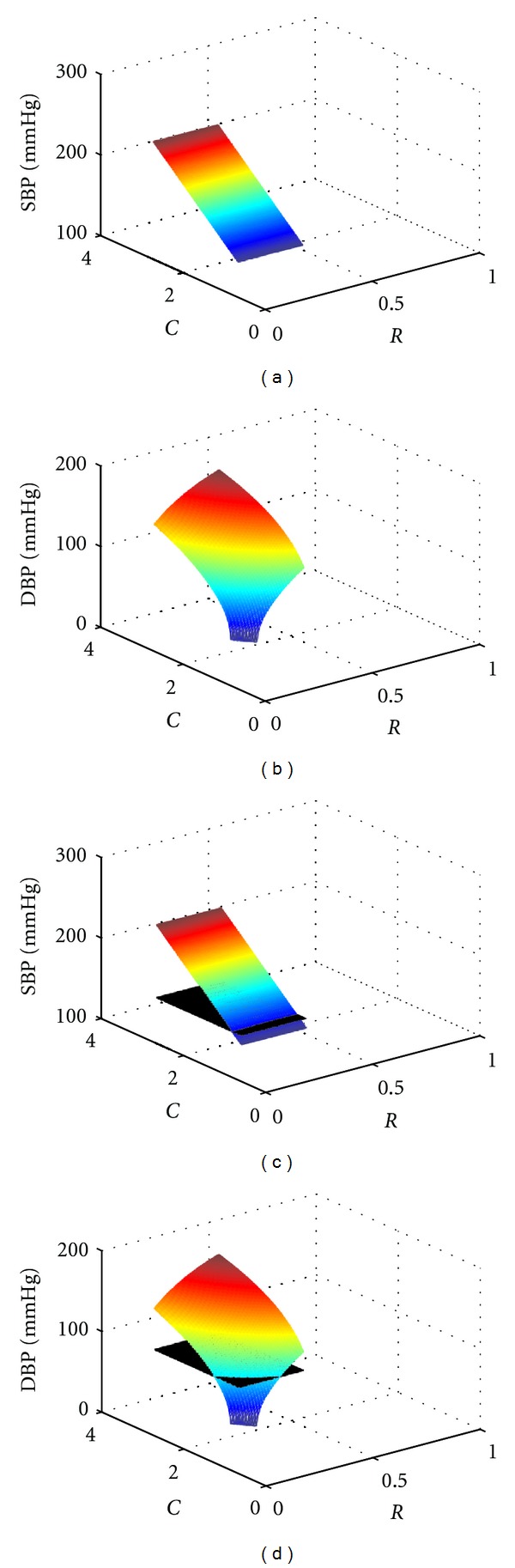
ISO surfaces of *RC* and blood pressure. (a) SBP-*RC* surface plotting. (b) DBP-*RC* surface plotting. (c) The plane of SBP = 120 mmHg intersects with SBP-*RC* surface. (d) The plane of DBP = 70 mmHg intersects with DBP-*RC* surface.

(4) Pi=K1·e−t(RC−R2C2−4CI)/2CI+K2·e−t(RC−R2C2−4CI)/2CI +{−2.8π×105(π2CI2−9I400+9R2C400)cos⁡⁡(20πt3)       +945Rsin⁡⁡(20πt3)+5670+1.12     ×107[π4C2I2−9200(−R2C2+I)Cπ2](Pi+135+R2)}   /[162+3.2π4C2I2×105    +(−1.44×104CI+7200R2C2)π2],

where *K*_1_and *K*_2_ are coefficients for general solution. According to Luo et al. [13], blood inertia *I* is set to 0.23 and the boundary value *P*_*i*+1_ is set to 30 mmHg. When *K*_1_ = 1∧*K*_2_ = 1, *P*_*i*_ reaches the maximum value at *t* = 0.14 s, so *P*_*i*_ (*t* = 0.14 s) is chosen to be the SBP. The expression of *P*_*i*_ (*t* = 0.14 s) is also a function of *R* and *C*. With reasonable ranges of *R* and *C* (*R* ∈ [1.55,3.60], *C* ∈ [0.30,0.60], suggested in Luo et al. [13]), a mapping data surface of SBP and *RC* is produced, as shown in Figure 3(a).

Blood flow *Q*_*i*−1_ in diastolic period (*t* ∈ [0.3, 0.8]) is 0. Solving (2), a general solution as the expression of blood pressure in diastolic period is derived as

(5) Pi=K3·e−t(RC−R2C2−4CI)/2CI+K4·e−t(RC−R2C2−4CI)/2CI +Pi+1,

where *K*_3_ and *K*_4_ are coefficients for the general solution. When *K*_3_ = −200∧*K*_4_ = −400, the waveform of the function has the maximum value at *t* = 0.4 s, then decreases monotonically till the minimum at *t* = 0.8. Such a waveform is considered to be a classic diastolic pressure wave, so the specific solution can be regarded as blood pressure in diastolic period and *P*(0.8) is chosen to be the DBP. The *P*_*i*_ (*t* = 0.8 s) is also a function of *R* and *C*. With the same reasonable range of *R* and *C* as in systolic period, the mapping data surface of DBP and *RC* is produced as shown in Figure 3(b).

**Figure 4:**
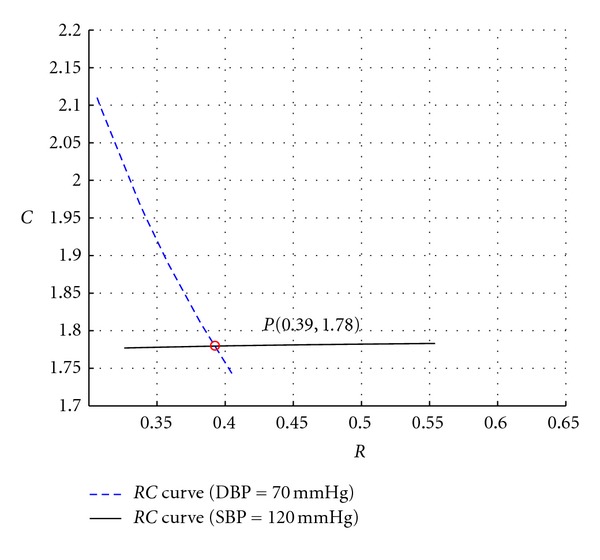
Solution of *R* and *C* under given SBP and DBP. The solid line is the relationship curve of *RC* in the case of SBP = 120 mmHg and the dotted line is the relationship curve of *RC* in the case of DBP = 70 mmHg. The red point *P* is the intersection of these two lines, indicating the solution of *RC* under SBP = 120 mmHg and DBP = 70 mmHg.

With SBP and DBP given, the solutions of *RC* can be directly derived from the data surface. For example, if one's SBP/DBP is 120/70 mmHg, a plane of *P*_*i*_ = 120 mmHg intersects with the SBP-*RC* surface in Figure 3(a), and an *RC* curve against SBP is obtained, as shown in Figure 3(c). In a similar manner, an *RC* curve against DBP is shown in Figure 3(d). When *RC* versus SBP function and *RC* versus DBP function are merged to an identical *R*-*C* plane, the intersection of them are the *R* and *C* values under SBP/DBP = 120/70 mmHg (Figure 4).

### 2.2. Simulation of HF Treatment Efficacy by ACEI

For simulation of ACEI treatment, two aspects are taken into account: the way ACEI affects hemodynamic state and the assessment of its effect.

It is known that ACEI poses effects mainly on the restoration of vascular property: resistance and compliance [14], so this study focuses on the change in *R* and *C* before and after ACEI treatment. By applying the fast parameters estimation algorithm, a unique solution of *RC* corresponding to HF patients' blood pressure can be attained. In order to obtain the change of *RC* under different treatment, we investigate 8 groups of patients with different doses of ACEI. The blood pressure records are from reported trials [15–19], whose baseline characteristics are shown in Table 1.

After the treatment of different doses of ACEI, SBP and DBP are reduced at different levels, leading to a new solution of *RC*. Δ*R* and Δ*C* denote the change percentages of *R* and *C*, and the subscripts pre/post denote the parameters before and after treatment:

(6) $$\begin{array}{c} \Delta R=\frac{R_{\text{post}}-R_{\text{pre}}}{R_{\text{pre}}}, \end{array}$$

(7) $$\begin{array}{c} \Delta C=\frac{C_{\text{post}}-C_{\text{pre}}}{C_{\text{pre}}}. \end{array}$$

Investigating Δ*R* and Δ*C* under different dose treatment helps understand the mechanism of ACEI in altering hemodynamic status. The behavior of vascular resistance and compliance can be depicted by producing a curve of Δ*R* and Δ*C* against dose of ACEI. As typical ACEI medicine, enalapril and lisinopril are considered in the study, and the comparison of their acting manner on *R* and *C* leads to better distinguishing diverse mechanisms of ACEI medications.

A cardiovascular system model can then be used to simulate drug efficacy at different doses.

The output parameters of the model are mean arterial pressure (MAP), pulse pressure (PP), heart rate (HR), cardiac output (CO), stroke volume (SV), ejection fraction (EF), stroke work (SW), and so on. These hemodynamic parameters are closely related to cardiac function and are vital for indicating the improvement or deterioration of heart failure. Regarding the outcome of the treatment, a comprehensive index, cardiac integrated index (CII), is produced to assess the hemodynamic state:

(8) $$\begin{array}{c} \text{CII}=\sum_{i=1}^{7}y_{i}\times w_{i}, \end{array}$$

where *y*_*i*_ is the value of output parameters and *w*_*i*_ is the weighting coefficient of output parameters determined by principal component analysis, as listed in Table 2. Positive weighting coefficient suggests that the smaller the value of the parameter is, the better the cardiac condition will be, so the reduction of CII value is a sign of patient's recovery.

## 3. Results

**Figure 5:**
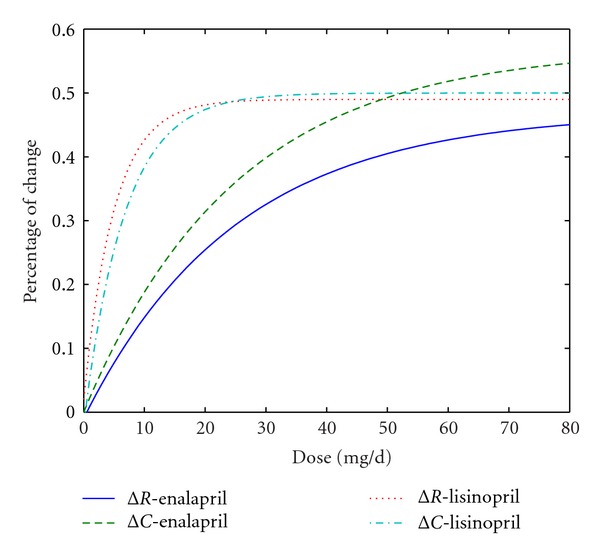
The trend curves of Δ*R* and Δ*C* under dose-dependant treatment of enalapril and lisinopril. The blue solid line is the trend curve of Δ*R* under different doses of enalapril; the green dotted line is the trend curve of Δ*C* under different doses of enalapril; the red dotted line is the trend curve of Δ*R* under different doses of lisinopril; the blue dotted line is the trend curve of Δ*C* under different doses of lisinopril.

By fast parameters estimation algorithm, the dose effect of common ACEIs, enalapril and lisinopril, on *R* and *C* is produced.  Figure 5 illustrates the change of *R* and *C* (Δ*R* and Δ*C* in percentage) under different ACEI doses. It is observed that for the lisinopril treatment Δ*R* and Δ*C* curves rise sharply and reach the saturation with a dose of 20 mg/d, and both curves run closely. In contrast, Δ*R* and Δ*C* under enalapril treatment change gradually but separately at each dose. In addition, for the doses below 40 mg/d, Δ*R* and Δ*C* induced by lisinopril are larger than those by enalapril, and the changes of *R* and *C* under ACEI treatment tend to be parallel with doses higher than 40 mg/d.

On the basis of ACEI dose and *R*/*C* paired data collected from [15–19], the dose-effect relationship curves are fitted. So the variation of *R*/*C* under arbitrary ACEI dose is determined. Drug action is simulated by adjusting the *RC* parameters in the cardiovascular model. Then the output values of SBP, DBP, MAP, PP, HR, CO, SV, and other parameters can be obtained. Following the process above, specific model is carried out. Just inputting the information of new patient into the model, the hemodynamic parameters can be estimated.

In this study, we applied the model to individual patients [20, 21] and predicted their outcome after medical therapy. The typical results of a subject are shown in Table 3, including the model output and the observed blood pressure in clinical trials. The baselines of hemodynamic parameter and CII before treatment are simulated until the estimated blood pressures are converged to the initial ones, as shown in column 2. The simulation is also done on effects of 20 mg/d of enalapril, and the predicted blood flow condition after drug treatment is shown in column 3. The measured values of SBP and DBP from patients taking 20 mg/d of enalapril are shown in column 4.

 It can be seen that the prediction of blood pressure approximates to the situation after treatment in clinical trials [20]. Comparing the hemodynamic parameters and CII of column 2 with those of column 3, we can observe a trend towards better situations. Decrease of blood pressure (SBP, DBP, MAP, and PP) shows the restoration of pressure regulation. The increase of CO, SV, and EF and decrease of SW indicate the enhancement of elasticity of heart muscles, promotion of the pumping function, and reduction of work by heart muscles. The decrease of CII is a sign of overall recovery of cardiac function. Even though there is no real measurement of the hemodynamic parameters (HR, CO, SV, EF, and SW), the simulation is successful on SBP and DBP. Similar results are verified by the simulation of lisinopril.

**Figure 6:**
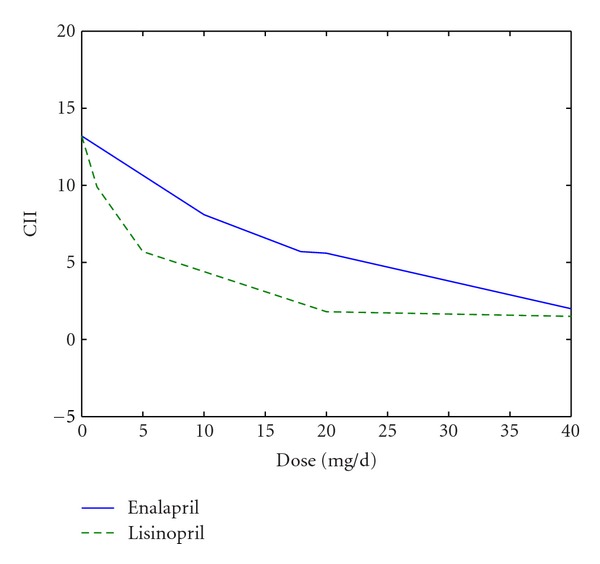
The CII at different doses. The solid line is the trend of patients' cardiac integrated index (CII) under different doses of enalapril; the dotted line is the trend of patients' CII under different doses of lisinopril.

CII is an integrated parameter representing the hemodynamic status. We studied its performance at different doses of medicine. Simulations on dataset from reference [21] under diverse doses of enalapril/lisinopril are accomplished to obtain hemodynamic parameters after treatment. CII is then derived from those parameters, as shown in Figure 6. The solid line represents the simulated CII under different doses of enalapril, while the dotted line is for lisinopril. In the figure, it is seen that CII does not drop significantly with dose of enalapril lower than 40 mg/d. The CII in lisinopril group falls rapidly when the dose <20 mg/d and then goes almost unchanged with the increase of dose. In general, the results reveal that with a raise of enalapril/lisinopril dose, the CII decreased. This means that the reduction of CII can be an indicator of the recovery of the patient's overall cardiac function. By observing the trends of CII curve, we can evaluate the different impacts of various drugs on cardiac function.

## 4. Discussion

In this paper, a new algorithm for fast estimation of vascular model parameters is presented. Vascular resistance and compliance that play important roles in the medical therapy for HF are calculated from the data surfaces constructed by SBP and DBP, which can be easily obtained from regular clinical examination. Compared with the previous method, which will take 2–5 hours to achieve the appropriate *RC* values, the present one is able to determine a unique approximation of *RC* considerably faster and more accurate. This novel method of parameter estimation can also be extended to the application in other mathematical physiological models.

Figure 5 shows the diverse mode of enalapril and lisinopril. Since Δ*R* is greater than Δ*C* at the same dose of enalapril, it suggests that enalapril affects the cardiac condition mainly by adjusting the vascular resistance. The Δ*R* and Δ*C* curves of lisinopril are close to each other indicating that lisinopril works by regulating *R* and *C* simultaneously. Therefore, we deduce that enalapril plays an important role in HF treatment mainly through relaxing blood vessel, since it significantly reduces the vascular resistance. Lisinopril exhibits effects in both dilating blood vessel and increasing vascular elasticity, contributing to changes to vascular resistance and vascular compliance.

The simulation results (Figure 6) imply the different patterns of efficacy between enalapril and lisinopril based on the various modes. The effect of enalapril on patients is mild and smooth, while the effect of lisinopril is rapid. It suggests that, at small doses, lisinopril has a more significant effect on improvement than enalapril. With doses higher than 40 mg/d, the two drugs' performances are similar. These differences result from the diverse effects of enalapril and lisinopril on Δ*R* and Δ*C*.

Similar results also have been disclosed in clinical trials and other studies. It has been reported that both ACEI drugs can regulate cardiac situation, and, within a certain range, the larger the dose is, the more amelioration can be seen in cardiac function [22]. Simpson and Jarvis, Menne et al., and Terpstra et al. [23–25] reported that lisinopril would result in a better improvement in HF due to its high tissue affinity, in contrast with enalapril. However, enalapril and lisinopril have a similar efficacy when their doses reach the highest approved level of the treatment [26]. An explanation may be that the binding of ACEI medicine and angiotensin-converting enzyme is a saturation reaction. Regular clinical dose is 10–20 mg for enalapril and 20–40 mg for lisinopril [27]. Our results show that, with 10–20 mg of enalapril or 20–40 mg of lisinopril, simulated blood pressure returns to normal or SBP/DBP decreases by 10 mmHg. Other simulated hemodynamic parameters (such as CO and EF) are also improved. The simulated effects meet the requirement of regular treatment [20].

The difference in the acting manner of ACEIs may provide hints in clinical practice. Our results suggest using lisinopril when patients need a rapid improvement in the physical condition. For acute HF, it is reasonable to use enalapril because it may give a smoother reduction in blood pressure with a lower risk of sudden hypotension. It is worth pointing out that enalapril can be a satisfactory agent for severe HF considering safety, which has been indicated by Dickstein et al. [28].

It is worth noting is that the developed model can also be applied to predict the effect of HF treatment individually. For this purpose, the blood pressure (SBP, DBP) of patients should be first measured and then the fast parameter estimation algorithm is utilized to obtain the corresponding baseline of the model parameters *R*/*C*. According to the drug effect curve, which describes the dose effect of ACEI on *R*/*C*, the variation of *R*/*C* under a given drug dose can be obtained. Finally, knowing the baseline and variation of *R*/*C*, we can apply the developed model to estimate the prediction of hemodynamic parameters and overall treatment effect. Referring to Table 3, the results demonstrate a match of predicted and observed blood pressure from reference [20, 21] within an error of 5%, giving proof of the reliability. Though the reference did not provide records of other hemodynamic parameters (such as CO, SV, and EF) measurement, these parameters can be obtained by model simulations. Then the comprehensive index CII will be calculated from them to reflect cardiac function. The predicted CII below the baseline may suggest a better prognosis after the treatment.

The simulation methods can be further used to evaluate other HF medication, that is, beta-blockade, diuretics, angiotensin receptor blocker, and so on. For instance, the drug efficacy of beta-blockade can be simulated by adjusting model parameters: decrease sympathetic nerve activity, increase vagus nerve activity as well as adjust blood volume. With the aiding of the model, the outcome of the treatment can be analyzed.

So far, the present HF simulation model, however, only provides a primary evaluation of ACEI drugs and has certain limitations. First, the model is not applicable to all patients because of the variety in individual reaction to medicines. The current cardiovascular model does not take into account the complicated circulation system. For instance, Sandoval et al. [29] reported that for certain HF patients undergoing lisinopril treatment, there is no significant improvement of blood pressure or cardiac index. The reason may be related to the cardiac antiadrenergic properties of those patients. Second, the applied data in this study are from the reported five large-scale clinical trials with different therapy periods and blood pressure levels, which may lead to bias in parameters estimation. Finally, the model reliability should be confirmed by more verification. We have evaluated the model by estimating the blood pressures, which approach the real ones. In future work, CO, SV, EF, and other hemodynamic parameters should be collected before/after treatments to verify the feasibility of the model. For improving and further validating the model performance, more clinical investigations are expected.

## 5. Conclusions

HF is a serious cardiovascular disease, which causes an increasing burden on public healthcare. Mathematical modeling and simulation in cardiovascular research have attracted much attention in the recent years. However, the difficulties in parameters estimation hinder the clinical applications of these models. This study presents a novel algorithm for fast estimation of the cardiovascular model parameters. Starting from the pathological parameter setting, the HF treatment can be simulated by adjusting vascular resistance and compliance. The dose effect is evaluated by comparing the model-derived blood pressure with the clinic measurement as well as a comprehensive index CII. The results demonstrate a 5% error between the simulated and measured blood pressure. In addition, we also obtained the CII index which can comprehensively reflect heart condition. We further applied this method to study the dose-effect relationship of ACEI medicine. A relationship curve is produced and the different outcome of enalapril and lisinopril can be distinguished. These results coincide with the conclusions from clinical trials and previously studies. Moreover, this work may offer a quantitative tool for constructing patient-specific treatment plans of HF treatment and can be used in evaluating the dose effect of other HF medications.

## Figures and Tables

**Table 1:** The baseline characteristics of 8 groups of HF patients.

Group	Size	Sex		Age in years	Blood pressure		Data source
		Male	Female		SBP (mmHg)	DBP (mmHg)	
1	122	98	24	57	118	78	Nanas et al., 2000 [15]
2	19	8	11	71	136	84	Louis et al., 2009 [16]
3	87	57	30	59	143	95	Bai and Wen, 2009 [17]
4	148	82	64	46	154	93	Hermida et al., 2008 [18]
5	41	38	3	58	158	100	Gomez et al., 1989 [19]
6	41	37	4	56	159	100	Gomez et al., 1989 [19]
7	44	42	2	54	158	102	Gomez et al., 1989 [19]
8	43	37	6	57	161	101	Gomez et al., 1989 [19]

**Table 2:** The output hemodynamic parameters of the model and weighting coefficients in CII.

Parameter	MAP	PP	HR	CO	SV	EF	SW
Weighting coefficient	0.2333	0.3084	0.0415	−0.3737	−0.3678	−0.3566	0.3266

MAP: mean arterial pressure; PP: pulse pressure; HR: heart rate; CO: cardiac output; SV: stroke volume; EF: ejection fraction; SW: stroke work.

**Table 3:** Comparison of hemodynamic parameters by simulation and clinical observation.

	Baseline	Simulated	Measured
	value	value	value
Enalapril 20 mg/d			
SBP (mmHg)	155*	143	137*
DBP (mmHg)	101*	92	88*
MAP (mmHg)	119	109	
PP (mmHg)	54	51	
HR (beat/min)	73	73	
CO (L/min)	5.26	5.48	
SV (mL)	72	75	
EF (%)	28.35	32.05	
SW (J/beat)	2.95	2.70	
CII	9.85	4.01	

Lisinopril 20 mg/d			
SBP (mmHg)	158^+^	141	140^+^
DBP (mmHg)	94^+^	90	88^+^
MAP (mmHg)	115	107	
PP (mmHg)	64	51	
HR (beat/min)	73	73	
CO (L/min)	5.11	5.62	
SV (mL)	70	77	
EF (%)	27.24	34.53	
SW (J/beat)	3.03	2.48	
CII	13.22	1.80	

*Data acquired from the literature [20]. ^+^Data acquired from the literature [21]. The rest data are estimated by models.
